# Exploring Factors that Shape Adherence to Technology-Based Cognitive Interventions

**DOI:** 10.1093/geroni/igab046.3064

**Published:** 2021-12-17

**Authors:** Erin Harrell, Nelson Roque, Walter Boot, Neil Charness

**Affiliations:** 1 University of Alabama, Tuscaloosa, Tuscaloosa, Alabama, United States; 2 University of Central Florida, University of Central Florida, Florida, United States; 3 Florida State University, Tallahassee, Florida, United States

## Abstract

A cognitive intervention study was conducted to explore methods to improve adherence to a technology-based cognitive intervention and uncover individual differences that predict adherence (N = 120). The study was divided into two phases: (1) in which participants were asked to follow a prescribed schedule of training that involved gamified neuropsychological tasks administered via a tablet, and (2), in which participants were asked to play as frequently as they wished. Positively and negatively framed messages about cognitive health were delivered via the software program, and measures of cognition, technology proficiency, self-efficacy, technology attitudes, and belief in the benefits of cognitive training were collected. We computed an aggregate measure of adherence during each of the two phases, as well as a measure of daily engagement. Across data modeling approaches, the finding was consistent: only during Phase 2, was there evidence that positively-framed messages encouraged greater adherence over negatively-framed messages. Measures of memory and self-efficacy demonstrated some, but limited, ability to predict individual differences in adherence.

